# *Nascer nas Casas de Parto do Brasil*: protocolo de pesquisa

**DOI:** 10.1590/0102-311XPT233023

**Published:** 2025-02-24

**Authors:** Nathalie Leister, Jamile Claro de Castro Bussadori, Ana Paula Esteves-Pereira, Silvana Granado Nogueira da Gama, Gisele Almeida Lopes, Maria Luiza Gonzalez Riesco, Nágela Cristine Pinheiro Santos, Bruna Dias Alonso, Nayara Ruiz Cintra, Maria do Carmo Leal, Christine McCourt

**Affiliations:** 1 City St. George’s, University of London, London, U.K.; 2 Escola de Enfermagem, Universidade de São Paulo, São Paulo, Brasil.; 3 Universidade Federal de São Carlos, São Carlos, Brasil.; 4 Escola Nacional de Saúde Pública Sergio Arouca, Fundação Oswaldo Cruz, Rio de Janeiro, Brasil.; 5 Escola de Enfermagem, Universidade Federal de Minas Gerais, Belo Horizonte, Brasil.; 6 Centro Universitário FAM, São Paulo, Brasil.; 7 Pesquisadora independente, São Paulo, Brasil.

**Keywords:** Centros de Assistência à Gravidez e ao Parto, Gravidez, Parto, Período Pós-parto, Enfermagem Obstétrica, Birthing Centers, Pregnancy, Parturition, Postpartum Period, Obstetric Nursing, Centros de Asistencia al Embarazo y al Parto, Embarazo, Parto, Período Posparto, Enfermería Obstétrica

## Abstract

Os Centros de Parto Normal (CPN) no Brasil são definidos como unidades de saúde vinculadas a um estabelecimento hospitalar, podendo estar localizados em suas dependências internas (CPN intra-hospitalares) ou imediações (CPN peri-hospitalares ou Casa de Parto). As estruturas física e assistencial das Casas de Parto são definidas pelo Ministério da Saúde. Fazem parte do seu modelo de cuidado as práticas adequadas, bons desfechos maternos e infantis e a promoção da experiência positiva da mulher no parto. O *Nascer nas Casas de Parto do Brasil* é um estudo transversal, em âmbito nacional, com os objetivos principais de avaliar a estrutura das Casas de Parto para atendimento a partos de risco habitual; avaliar a assistência ao parto e nascimento nas Casas de Parto; comparar os resultados desta pesquisa com os de nascimentos de risco habitual ocorridos nos hospitais amostrados na pesquisa *Nascer no Brasil II*. Este artigo tem como objetivo informar o delineamento e as etapas metodológicas propostas para o *Nascer nas Casas de Parto do Brasil*. Foram selecionadas oito Casas de Parto e fazem parte da amostra as gestoras dos serviços e 1.560 mulheres usuárias. A coleta de dados compreende o período de agosto de 2022 a outubro de 2024. Apresentam-se as variáveis do estudo e instrumentos, as etapas de coleta de dados desde a admissão até 120 dias após o parto, as atividades da equipe de pesquisa, o plano de análise dos dados e os aspectos éticos. Discute-se a relevância da divulgação deste protocolo de pesquisa e as especificidades de pesquisa em Casas de Parto.

## Abstract (en)

In Brazil, Freestanding Birth Centers (FBC) are defined as health units linked to hospitals that may be located within these facilities (alongside birth center - ABC) or in their surroundings (FBC). The Brazilian Ministry of Health has defined the physical and care structure of FBC. Their care model includes appropriate practices, good maternal and infant outcomes, and the promotion of positive experiences for women during childbirth. *Births in Brazilian Birthing Centers* is a nationwide cross-sectional study aimed to evaluate the structure of FBC to care for usual births risks, assess labor and birth care in FBC, and compare the research outcomes with those of usual births risks in the hospitals within *Birth in Brazil II*. This article aims to inform the design and methodological steps proposed for *Births in Brazilian Birthing Centers*. In total, eight FBC were chosen. The sample included service managers and 1,560 female users. Data collection were collected from August 2022 to October 2024. Study variables and instruments, the stages of data collection from admission up to 120 days after childbirth, the research team’s activities, the data analysis plan, and the ethical aspects are shown and the relevance of disseminating this research protocol and the specificities of research in Birthing Centers are discussed.

## Abstract (es)

Los Centros de Parto Normal (CPN) en Brasil se definen como centros de salud vinculados a un hospital y pueden estar situados en su interior (CPN intrahospitalarios) o en las cercanías (CPN perihospitalarios o Casa de Parto). La estructura física y asistencial de las Casas de Parto está definida por el Ministerio de Salud. Las prácticas adecuadas, los buenos resultados maternoinfantiles y la promoción de la experiencia positiva de la mujer en el parto forman parte de su modelo de atención. *Nacer en Casas de Parto en Brasil* es un estudio transversal, a nivel nacional, con los siguientes objetivos: evaluar la estructura de las Casas de Parto en cuanto a la atención de partos de riesgo habitual; evaluar el parto y la asistencia a este procedimiento en Casas de Parto; y comparar los resultados de esta investigación con los de los nacimientos de riesgo habitual que tuvieron lugar en los hospitales participantes en la encuesta *Nacer en Brasil II*. Este artículo tiene como objetivo informar el diseño y los pasos metodológicos propuestos para *Nacer en Casas de Parto en Brasil*. Se seleccionaron ocho Casas de Parto, y la muestra estuvo compuesta por las gestoras de los servicios y 1.560 usuarias. La recopilación de datos abarca el período comprendido entre agosto de 2022 y octubre de 2024. Se presentan las variables e instrumentos del estudio, las etapas de recolección de datos desde el ingreso hasta 120 días después del parto, las actividades del equipo de investigación, el plan de análisis de datos y los aspectos éticos. Se discute la relevancia de la divulgación de este protocolo de investigación y los detalles de la investigación en Casas de Parto.

## Introdução

No Brasil, a maioria dos partos e nascimentos ocorre nos hospitais, onde a assistência usualmente é realizada com intervenções rotineiras e, portanto, nem sempre necessárias. Esse cenário de assistência hospitalar e intervencionista é questionado devido ao constante aumento do número de cesarianas e de denúncias de casos de violência obstétrica ^1^.

Desde a década de 1990, vem-se formulando políticas públicas com potencial para melhorar a assistência às gestantes e aos bebês, especialmente no parto e nascimento. Entre elas, houve a criação dos Centros de Parto Normal (CPN) no âmbito do Sistema Único de Saúde (SUS) ^2^.

No início dos anos 2000, havia alguns CPN em funcionamento no país, porém, foi com a estratégia federal Rede Cegonha, em 2011, que esse modelo ganhou mais destaque, com incentivo, construção, ampliação, aquisição de equipamentos e custeio mensal de CPN ^3^. Atualmente, os CPN no Brasil estão definidos como unidades de saúde vinculadas a um estabelecimento hospitalar, localizada em suas dependências internas ou imediações. De acordo com sua localização em relação ao hospital de referência, ele pode ser classificado como intra-hospitalar (CPNi) ou peri-hospitalar (CPNp) - também conhecido como Casa de Parto ^4^.

Os CPN oferecem um ambiente seguro, confortável e acolhedor ^5^ ^,^ ^6^ ^,^ ^7^, estimulam a autonomia e escolha informada da mulher, facilitam uma transição positiva para o período pós-parto ^7^ ^,^ ^8^ ^,^ ^9^ e têm o potencial de promover a mudança do modelo de cuidado vigente para o modelo de cuidado biopsicossocial ^7^ ^,^ ^8^ ^,^ ^10^. Adotando um conjunto de práticas baseadas em evidências científicas - incluindo a liderança do cuidado por obstetrizes e enfermeiras obstétricas ^11^ ^,^ ^12^ - os primeiros estudos brasileiros nesses locais indicavam menores taxas de intervenções, maior uso de boas práticas assistenciais e melhores índices de satisfação quando comparados aos centros obstétricos ^5^. Esses resultados se reafirmam em estudos recentes ^13^ ^,^ ^14^ que também apontam os CPN como menos onerosos que os centros obstétricos ^13^ ^,^ ^14^ ^,^ ^15^ ^,^ ^16^ ^,^ ^17^ ^,^ ^18^ ^,^ ^19^.

Embora esse modelo funcione há mais de duas décadas no Brasil, não existem pesquisas de abrangência nacional sobre as Casas de Parto. *Nascer no Brasil II: Pesquisa Nacional sobre Aborto, Parto e Nascimento* representa uma possibilidade para conhecer também a assistência prestada nos estabelecimentos de saúde localizados fora do ambiente hospitalar e disponíveis no SUS, ou seja, as Casas de Parto. Nesse sentido, o estudo denominado *Nascer nas Casas de Parto do Brasil* está integrado à pesquisa *Nascer no Brasil II*, em andamento ^20^.

Preliminarmente, foram estabelecidas as hipóteses que balizaram os objetivos do projeto *Nascer nas Casas de Parto do Brasil*. Considerando as características do modelo assistencial biopsicossocial das Casas de Parto, essas hipóteses foram: (1) a estrutura física e assistencial das Casas de Parto está de acordo as diretrizes do Ministério da Saúde; (2) as boas práticas de assistência ao parto e pós-parto associam-se com melhores desfechos maternos e infantis; (3) as transferências de cuidado da Casas de Parto para o hospital de referência ocorrem de maneira oportuna e não repercutem negativamente nos desfechos maternos e neonatais; (4) iniciar o atendimento ao trabalho de parto nas Casas de Parto diminui as taxas de cesariana; (5) a satisfação materna com o parto e com o atendimento recebido é alta entre as mulheres que foram atendidas em Casas de Parto; (6) as taxas de desrespeitos e abusos ocorridos durante a assistência ao parto e pós-parto em Casas de Parto é baixa.

Assim, dadas as especificidades das Casas de Parto, este artigo tem como objetivo informar o delineamento e as etapas metodológicas propostas para o *Nascer nas Casas de Parto do Brasil*.

## O estudo *Nascer nas Casas de Parto do Brasil*

### Objetivos

Os objetivos gerais são: (1) avaliar a estrutura, organização e indicadores de assistência ao parto das Casas de Parto; (2) avaliar as práticas e os resultados da assistência ao parto e nascimento nas Casas de Parto; (3) comparar os resultados deste estudo com os de nascimentos de risco habitual ocorridos nos hospitais amostrados no *Nascer no Brasil II*.

### Desenho

Estudo transversal, de abrangência nacional, integrado à pesquisa *Nascer no Brasil II*.

### Local

Fazem parte do estudo oito Casas de Parto e seus respectivos hospitais de referência, localizados nos estados de São Paulo (n = 2), Bahia (n = 1), Distrito Federal (n = 1), Minas Gerais (n = 1), Pará (n = 1), Pernambuco (n = 1) e Rio de Janeiro (n = 1). Os hospitais de referência foram incluídos na pesquisa para seguimento dos casos de transferência intraparto, pós-parto e neonatal.

Para a inclusão na pesquisa, a Casa de Parto deve ter atendido mais de 100 partos no ano de 2019, segundo o Sistema de Informações sobre Nascidos Vivos (SINASC). Esse critério foi utilizado levando em consideração a logística e capacidade da equipe de pesquisa para coletar dados presencialmente em áreas remotas, onde se localizam Casas de Parto com menos de 100 partos/ano e com baixo fluxo de atendimento; e igualar aos critérios de inclusão de estabelecimentos de saúde do *Nascer no Brasil II*, que incluiu na amostra de estabelecimentos, somente hospitais que atenderam mais de 100 partos no ano de 2019.

No ano de 2020, foram consultados os estabelecimentos que constavam no Cadastro Nacional de Estabelecimentos de Saúde (CNES) como CPNp ou CPN isolado e foram obtidos os seguintes resultados:

(a) Código 61 - Tipo de estabelecimento: CPNp isolado ^2^ = 20 estabelecimentos;

(b) Código 1412 - Habilitação: CPNp com cinco quartos PPP (pré-parto, parto e pós-parto) ^4^ = 7 estabelecimentos;

(c) Código 1417 - Habilitação: CPNp com três quartos PPP ^4^ = 1 estabelecimento.

Desses 28 estabelecimentos, três estavam cadastrados em duplicidade, 13 foram confirmados como Casa de Parto e apenas oito atenderam mais de 100 partos no ano de 2019 ^21^, os quais foram considerados elegíveis para o *Nascer nas Casas de Parto do Brasil* e incluídos na pesquisa.

### População

A população do *Nascer nas Casas de Parto do Brasil* é constituída por profissionais que atuam nas Casas de Parto (gestor, coordenador, líder etc.) e por gestantes admitidas para o parto sem dificuldade de entender a língua portuguesa. O critério de inclusão conflitante com o *Nascer no Brasil II* são as gestantes de alto risco, que serão excluídas na comparação com *Nascer nas Casas de Parto do Brasil*.

Os critérios de exclusão são: evadir-se da Casa de Parto ou hospital antes da alta; ser transferida ou ter seu bebê transferido para outro hospital que não seja de referência da Casa de Parto; fazer entrega legal do bebê.

### Amostra

Para o cálculo amostral, foram consideradas: (1) a estimativa de 82% de partos vaginais de gestantes usuárias das Casas de Parto e a prevalência de 77% obtida no estudo *Nascer no Brasil I* ^22^ entre as gestantes de risco habitual dos hospitais do SUS, também estimada para o *Nascer no Brasil II*.

Para detectar uma diferença de pelo menos 5% nessa prevalência, a amostra calculada foi de 1.560 mulheres (já considerando a estimativa de 25% transferências intraparto das Casas de Parto ^7^).

Esta amostra tem também 90% de poder para detectar aumento de 6% nos desfechos de magnitude de 50% (por exemplo, boas práticas na atenção ao parto) no *Nascer no Brasil II* em relação ao *Nascer nas Casas de Parto do Brasil* (50% e 56%), assim como para detectar redução de 2% para desfechos mais raros, como a internação em Unidade de Terapia Intensiva Neonatal (de 5% no *Nascer no Brasil II* para 3% no *Nascer nas Casas de Parto do Brasil*). Sendo assim, o tamanho amostral se mostra suficiente para as comparações dos desfechos secundários.

O nível de significância utilizado foi de 5% e o poder de 90%.

O número amostrado foi distribuído proporcionalmente entre as Casas de Parto, de acordo com o número de partos de 2019 (Tabela 1).

**Tabela 1:** Número de partos em 2019 e amostra no *Nascer nas Casas de Parto do Brasil*.

Casa de Parto	Partos em 2019	Amostra por Casa de Parto
Casa de Parto 1	886	468
Casa de Parto 2	639	337
Casa de Parto 3	408	215
Casa de Parto 4	407	215
Casa de Parto 5	211	113
Casa de Parto 6	153	81
Casa de Parto 7	130	69
Casa de Parto 8	118	62
Total	2.952	1.560

### Variáveis do estudo e instrumentos

As variáveis do estudo estão detalhadas nos instrumentos de coleta de dados, que são seis questionários fechados, adotados pelo *Nascer no Brasil II* e que para este estudo foram adaptados textualmente para contemplar as especificidades de atendimento ao parto em Casas de Parto. A adaptação textual foi cuidadosamente feita para garantir a comparabilidade entre os resultados dos estudos *Nascer no Brasil II* e *Nascer nas Casas de Parto do Brasil*, contemplando um dos objetivos deste estudo. Os instrumentos adaptados estão disponíveis no *site* https://nascernobrasil.ensp.fiocruz.br/?us_portfolio=nascer-no-brasil-2.

A seguir, são apresentados os seis instrumentos de coleta de dados e os dados que são coletadas por meio de cada um deles.

#### Instrumento da entrevista com profissionais

Inclui os dados sobre a identificação, caracterização e funcionamento da Casa de Parto: serviços e informações disponíveis, comissões e estrutura física; relacionamento com o usuário; equipamentos assistenciais de uso diário e para atendimento de urgências e emergências; medicamentos disponíveis; recursos humanos, capacitação, organização e processo de trabalho (protocolos em uso e formas de disponibilização e acesso); critérios de admissão; indicadores assistenciais ^4^; formas de divulgação do estabelecimento para a população; capacidade instalada de leitos; adaptações no oferecimento de serviços durante a pandemia de COVID-19.

#### Instrumento da entrevista com a mulher na Casa de Parto ou no hospital de referência

Inclui dados de caracterização das mulheres admitidas para o parto: identificação; escolaridade; renda; antecedentes clínicos e obstétricos; dados antropométricos; plano de saúde; contexto da gestação atual; assistência pré-natal; morbidades e medicações utilizadas durante a gestação; trabalho de parto e parto; acompanhante; intenção de amamentar; informações recebidas durante o pré-natal sobre direitos, o parto, o bebê e aleitamento materno; breve avaliação da assistência recebida.

#### Instrumento do cartão de pré-natal e ultrassonografias

Inclui dados sobre a assistência pré-natal: antecedentes clínicos e obstétricos; história obstétrica atual; número de consultas de pré-natal; exames clínicos e obstétricos realizados na consulta; resultados de exames laboratoriais; prescrição de suplementações nutricionais e medicamentos; vacinações; diagnósticos e prescrição de tratamentos de doenças ou intercorrências durante a gestação; idade gestacional, peso e características fetais constantes nas ultrassonografias ^23^ ^,^ ^24^.

#### Instrumento dos prontuários da mulher e do recém-nascido

Inclui dados dos prontuários da mulher e do recém-nascido das Casas de Parto e dos hospitais de referência (em casos de transferências): admissão; práticas assistenciais; intervenções realizadas ^25^; medicamentos administrados; morbimortalidade; internação em unidade de cuidado intermediário ou intensivo; condições na alta; registro de causa em casos de óbito.

#### Instrumento de entrevista com a mulher aos dois meses pós-parto

Inclui dados sobre o pós-parto: amamentação; morbimortalidade; utilização de serviço de saúde após a alta; vínculo com o bebê; saúde mental perinatal. Nessa entrevista, para coleta de alguns dados, também são adotados os seguintes instrumentos validados: *Escala Municipal de Trauma de Nascimento* (*City Birth Trauma Scale -* City BiTS) ^26^ para transtorno de estresse pós-traumático (TEPT); *Escala de Depressão Pós-natal de Edimburgo* (*Edinburgh Postnatal Depression Scale -* EPDS) ^27^ para depressão pós-parto; *Transtorno de Ansiedade Generalizada* (*Generalized Anxiety Disorder* - GAD-7) ^28^ para ansiedade; *Questionário de Vínculo Pós-parto* (*Postpartum Bonding Questionnaire* - PBQ) ^29^ para vínculo entre o binômio.

#### Instrumento de entrevista com a mulher aos quatro meses pós-parto

Inclui dados sobre o pós-parto: amamentação; morbimortalidade; utilização de serviço de saúde após a alta; satisfação com o atendimento e percepção sobre violência obstétrica, maus-tratos e abusos durante a internação para parto ^30^ (adoção de instrumento específico adaptado ^31^).

### Coleta de dados

A coleta de dados é realizada em quatro etapas e foi iniciada em 17 de agosto de 2022, com previsão de finalização em 31 de outubro de 2024. Para a coleta de dados, é utilizada a plataforma eletrônica REDCap (https://redcapbrasil.com.br/). Cada pesquisador tem seu próprio registro na plataforma e dispõe de *tablet* ou celular com internet e aplicativo do REDCap instalado.

#### Etapa 1

Entrevista presencial ou virtual com profissionais para coleta de dados relativos à identificação, caracterização e funcionamento da Casa de Parto. O pesquisador entra em contato com o gestor da instituição e agenda entrevista presencial ou virtual, de acordo com a preferência do entrevistado.

#### Etapa 2

Entrevista presencial com mulheres que deram à luz na Casa de Parto ou no hospital de referência após transferência intraparto. A entrevista é realizada antes da alta. Nessa etapa, também são realizadas a extração de dados do cartão de pré-natal e dos prontuários na Casa de Parto (e no hospital referência em caso de transferência materna intraparto e pós-parto ou transferência neonatal).

Antes da abordagem da mulher, o pesquisador deve identificar todas as mulheres admitidas em trabalho de parto e elaborar uma lista, por ordem de admissão, de abordagens para o dia. Todas as mulheres admitidas devem constar na lista, independentemente se deram à luz na Casa de Parto, foram transferidas ou tiveram o bebê transferido para o hospital. Nessa lista, são registrados: código da mulher na pesquisa; aceite, recusa, inelegibilidade ou perda; data e hora da internação na Casa de Parto (e no hospital, em casos de transferências); nome da participante; data e hora do parto; desfecho de nascimento do bebê (nascido vivo, natimorto ou neomorto); data e hora da entrevista; número dos prontuários; nome do coletador de dados.

Ao abordar uma mulher e convidá-la a participar de pesquisa, o pesquisador registra o aceite, recusa, inelegibilidade ou perda. Em caso de aceite, realiza-se a entrevista, registrando as respostas no REDCap. Em caso de recusa ou perda, a participante é substituída por outra com mesmo desfecho de transferência e de nascimento do bebê. Ou seja, se a perda ou recusa for uma transferência pós-parto, é substituída por outra mulher transferida após o parto. O mesmo desfecho do bebê, se a perda ou recusa for de uma mulher que teve um natimorto ou neomorto, ela é substituída por outra mulher que teve um natimorto ou neomorto.

#### Etapas 3 e 4

Entrevistas telefônicas com a mulher ou autopreenchimento de questionário, entre 45 e 60 dias pós-parto e entre 100 e 120 dias pós-parto. O pesquisador entra em contato com a mulher por telefone, mensagem de texto ou WhatsApp, que foram coletados na Etapa 1, e pergunta se ela gostaria de responder ao questionário por ligação telefônica ou por autopreenchimento. Se a escolha for o autopreenchimento, é gerado um *link* para o questionário e enviado à participante. Se for por ligação telefônica, a entrevista se inicia ou é marcada para outro dia e horário de conveniência da mulher; e o pesquisador realiza a entrevista, registrando as respostas no REDCap.

### Equipe de trabalho

A equipe de trabalho do *Nascer nas Casas de Parto do Brasil* é composta por duas coordenadoras que gerem uma equipe central, oito equipes de coleta de dados locais - uma equipe em cada Casa de Parto - e duas equipes de coleta dos dados pós-parto, das Etapas 3 e 4.

A equipe central atua em parceria com a equipe de pesquisa do *Nascer no Brasil II* e tem a responsabilidade de cumprir com demandas administrativas, gestão de projeto e de pessoas, anuência dos serviços e processos éticos; ser referência de contato e interlocução com os gestores das Casas de Parto e hospitais de referência; administrar, prover insumos e sanar dúvidas das equipes; recrutar coletadores de dados para comporem as equipes locais e de pós-parto; administrar, em conjunto com a equipe do *Nascer no Brasil II*, treinamento virtual e presencial das equipes; monitorar a coleta e os dados - escala de coletadores e manutenção do campo; gerir a plataforma REDCap; analisar a qualidade dos dados coletados e coletar os dados da Etapa 1.

As equipes locais têm a responsabilidade de compor e cumprir a escala diária de trabalho; identificar as puérperas para compor a amostra do estudo; cumprir com processos éticos; preencher os instrumentos de coleta de dados no REDCap, entrevistando as puérperas, coletando dados dos prontuários, dos cartões de pré-natal e dos laudos das ultrassonografias.

As equipes de coleta pós-parto têm a responsabilidade de entrar em contato com as mulheres nos períodos de realização das Etapas 3 e 4; cumprir com processos éticos e preencher os instrumentos de coleta de dados no REDCap ou enviar *link* para autopreenchimento dos questionários, conferindo, posteriormente, se foram preenchidos.

### Treinamento da equipe local e de pós-parto

Antes de iniciar a coleta de dados, todos os coletadores das equipes locais participam de treinamento com objetivo de padronizar o processo de recrutamento, abordagem e aplicação dos instrumentos, além de treinar o uso do REDCap.

O treinamento engloba a apresentação das pesquisas *Nascer no Brasil II* e *Nascer nas Casas de Parto do Brasil*, das atividades, responsabilidades e conduta ética, além do processo de envio de dados ao servidor da Fundação Oswaldo Cruz (FIOCRUZ) por meio do REDCap. São simuladas a identificação das mulheres admitidas para atendimento nas Casa de Parto; elaboração de lista de abordagem por ordem de admissão; entrevista presencial; extração de dados de prontuários, cartões de pré-natal e ultrassonografias; entrevista via telefone e envio de *link* autopreenchível. Os exercícios são corrigidos e retornados aos coletadores. O treinamento prático de quem coleta dados na Etapa 2 é realizado na própria Casa de Parto e de quem coleta dados nas Etapas 3 e 4, é realizado virtualmente pela plataforma Google Meet (https://meet.google.com).

### Controle da qualidade e monitoramento dos dados

Para assegurar a qualidade dos dados, a equipe central faz diariamente o monitoramento dos dados por meio do REDCap, além estar sempre disponível para o esclarecimento de dúvidas relacionadas à plataforma eletrônica, coleta ou nomenclatura dos instrumentos, prontuários, cartões de pré-natal e laudos de ultrassonografia.

### Análise dos dados

Os dados são armazenados no REDCap e exportados diretamente aos softwares de análise. Serão analisados com uso dos softwares SPSS 22.0 (https://www.ibm.com/) e R, versão 3.5.1 (http://www.r-project.org).

Os dados sobre a estrutura, organização, funcionamento e indicadores assistenciais das Casas de Parto serão analisados de forma descritiva e cotejados, tendo como referência as diretrizes estabelecidas na *Portaria nº 11/2015* ^4^ e na *RDC nº 36/2008* ^32^.

As características sociodemográficas, clínicas e obstétricas das mulheres e os dados da assistência pré-natal serão analisados de forma descritiva. Para as variáveis contínuas, serão calculados a média e o desvio padrão (DP) e para as variáveis categóricas, serão calculadas as frequências absoluta e relativa.

As práticas na assistência ao parto e nascimento e os resultados ou desfechos maternos e neonatais no parto e pós-parto serão analisados de forma descritiva e inferencial. O planejamento inicial da análise inferencial inclui os testes qui-quadrado ou teste exato de Fisher, para associar as variáveis categóricas com os desfechos, os testes t ou Wilcoxon-Mann-Whitney para associar as variáveis contínuas com os desfechos e a análise de variância (ANOVA) ou teste de Kruskal-Wallis, quando indicados.

Será adotado um modelo de regressão logística para controle de confundimento. A análise será ajustada, calculando-se o *odds ratio* (OR) e o respectivo intervalo de 95% de confiança (IC95%). Todos os testes serão realizados de forma bicaudal e será adotado o erro tipo I de 5% (valor de p = 0,05).

Para comparação com os resultados do *Nascer no Brasil II*, será realizada análise inferencial. Os modelos de analíticos definidos serão aplicados com apoio da equipe estatística responsável pela análise do *Nascer no Brasil II*.

## Aspectos éticos

Foi realizado contato com todas as oito Casas de Parto e os oito hospitais de referência para apresentar a pesquisa. Em alguns desses serviços, o contato e a apresentação foram feitos de forma presencial e, em outros, de forma remota, pelo Google Meet. Todos os estabelecimentos aceitaram participar da pesquisa.

Além da aprovação pelo Comitê de Ética em Pesquisa (CEP) da Escola Nacional de Saúde Pública Sergio Arouca (ENSP/FIOCRUZ; parecer nº 5.192.349), alguns serviços solicitaram aprovação dos próprios CEP, que foram considerados coparticipantes.

Para participar da pesquisa, é necessário assinar o Termo de Consentimento Livre e Esclarecido. Para menores de idade, também é necessária a assinatura do Termo de Assentimento pelo responsável legal.

## A relevância da divulgação do protocolo de pesquisa

O protocolo de pesquisa constitui passo preliminar essencial na realização de qualquer estudo. Consiste na exposição detalhada do método científico para responder às perguntas formuladas pelos pesquisadores ^33^.

A publicação deste protocolo de pesquisa favorecerá a transparência, acessibilidade e reprodutividade do estudo, aspectos recomendados como boas práticas científicas ^34^ e que se alinham ao compromisso da ciência com a verdade e com a população ^35^.

Neste artigo, demos ênfase às especificidades das Casas de Parto e sua população, o que justifica a adaptação dos questionários do *Nascer no Brasil II*. Por exemplo, para que seja possível a existência de uma Casas de Parto, é necessário convênio com hospital de referência para transferência de cuidados das mulheres e/ou seus recém-nascidos. Além disso, ainda em pequena quantidade no Brasil e representando menos de 1% de todos os nascimentos do país ^21^, não foi preciso cálculo amostral de estabelecimentos, pois todas as com mais de 100 partos/ano foram convidados e concordaram em participar da pesquisa.

A divulgação dos questionários, do delineamento e das etapas metodológicas da desta pesquisa, com o máximo detalhamento, permite a apreciação crítica da qualidade do estudo, bem como da validade dos resultados, quando de sua divulgação futura.

Diferentemente dos ensaios clínicos, que devem ter o protocolo registrado em alguma plataforma reconhecida nacional e internacionalmente, os estudos observacionais prescindem do registro prévio do protocolo de pesquisa. No entanto, para considerar a escrita deste protocolo, foram utilizadas as diretrizes para o relato de estudos observacionais STROBE (*Strengthening the Reporting of Observational Studies in Epidemiology*) ^36^.

## Considerações finais

A despeito das políticas públicas vigentes no Brasil e do lançamento das diretrizes nacionais de assistência ao parto normal ^37^, em 2016, os indicadores de assistência e saúde mostram desfechos maternos e perinatais negativos, reflexo da baixa qualidade da atenção obstétrica.

Consideramos que o país necessita, com urgência, transformar o modelo de assistência na gravidez e parto, promovendo e executando as práticas recomendadas e baseadas em evidências para aprimorar a qualidade do atendimento à população e avançar na melhoria dos desfechos de morbimortalidade materna e neonatal. Distinguimos as Casas de Parto como serviços potencializadores dessa melhoria, pois têm princípios e valores centrais da assistência baseada no modelo de cuidado biopsicossocial ^7^.

Além da segurança da assistência oferecida, esse modelo visa favorecer a experiência positiva no parto às mulheres e famílias, pois segundo a Organização Mundial da Saúde (OMS) ^25^, além de oferecer cuidados clinicamente eficazes, é necessário fazer mais para que as mulheres se sintam seguras e confortáveis com a experiência de parto e nascimento, englobando os cuidados holísticos e visando o entendimento integral do ser humano.

Ressaltamos que os acordos e protocolos de transferência adotados juntamente com o hospital de referência, são aspectos inerentes à existência das Casas de Parto. Nesse sentido, o *Nascer nas Casas de Parto do Brasil* também acompanha os desfechos das mulheres e bebês após transferência, componente que comumente não é estudado nas pesquisas brasileiras desenvolvidos nesses locais.

Portanto, com este estudo esperamos ampliar o conhecimento sobre diferentes locais para dar à luz no país, oferecidos pelo sistema público de saúde. A parceria com o *Nascer no Brasil II* possibilitará a comparação dos resultados das Casas de Parto com os dos hospitais, porque para isso será selecionada amostra de hospitais e de mulheres equiparáveis às que foram incluídas no *Nascer nas Casas de Parto do Brasil*. Além disso, para os desfechos principais, ambos os estudos têm as mesmas variáveis e coletam os mesmos dados até quatro meses após o parto. Vale ressaltar que o *Nascer nas Casas de Parto do Brasil*, diante da característica dos locais, de atender gestantes de risco habitual, não engloba o estudo dos casos de aborto.

Ademais, vale destacar o ineditismo dos resultados sobre a saúde mental relacionada ao parto, utilizando instrumentos validados para a avaliação do TEPT, ansiedade, depressão pós-parto e vínculo com o bebê, bem como um instrumento baseado na recomendação da OMS para prevenção e eliminação de abusos, desrespeito e maus-tratos durante o parto em instituições de saúde ^30^. Por fim, com esta investigação, esperamos contribuir para a escolha consciente e informada das mulheres sobre o local de parto.

## References

[1] Diniz CSG, Rattner D, Oliveira AFPL, Aguiar JM, Niy DY (2018). Disrespect and abuse in childbirth in Brazil social activism,
public policies and providers' training. Reprod Health Matters.

[2] Ministério da Saúde (1999). Portaria nº 985, de 5 de agosto de 1999. Cria o centro de parto
normal-CPN, no âmbito do Sistema Único de Saúde.. Diário Oficial da República Federativa do Brasil.

[3] Ministério da Saúde (2011). Portaria nº 1.459, de 24 de junho de 2011. Institui, no âmbito do
Sistema Único de Saúde - SUS - a Rede Cegonha.. Diário Oficial da União.

[4] Ministério da Saúde (2015). Portaria nº 11, de 7 de janeiro de 2015. Redefine as diretrizes
para implantação e habilitação de Centro de Parto Normal (CPN), no âmbito do
Sistema Único de Saúde (SUS), para o atendimento à mulher e ao recém-nascido
no momento do parto e do nascimento, em conformidade com o Componente PARTO
E NASCIMENTO da Rede Cegonha, e dispõe sobre os respectivos incentivos
financeiros de investimento, custeio e custeio mensal.. Diário Oficial da União.

[5] Riesco MLG, Oliveira SMJV, Bonadio IC, Schneck CA, Silva FMB, Diniz CSG (2009). Centros de Parto no Brasil: revisa~o da produc¸a~o
cienti´fica.. Rev Esc Enferm USP.

[6] Walsh D, Newburn M (2002). Towards a social model of childbirth part one. Br J Midwifery.

[7] Aguiar CA, Lopes GA, Bussadori JCC, Leister N, Riesco MLG, Alonso BD (2025). Modelo de atenção em Centros de Parto Normal peri-hospitalares
brasileiros: uma revisão de escopo.. Ciênc Saúde Colet.

[8] Rocca-Ihenacho L, Batinelli L, Thaels E, Rayment J, Newburn M, McCourt C (2018). Midwifery unit standards.

[9] McCourt C, Rance S, Rayment J, Sandall J, J (2011). Birthplace qualitative organisational case studies: how maternity care
systems affect the provision of care in different settings. Birthplace in
England research programme: final report part 6.

[10] Ministério da Saúde (2012). Manual prático para implementação da Rede Cegonha.

[11] Narchi NZ, Cruz EF, Gonçalves R (2013). O papel das obstetrizes e enfermeiras obstetras na promoc¸a~o da
maternidade segura no Brasil.. Ciênc Saúde Colet.

[12] Gama SGN, Viellas EF, Medina ET, Angulo-Tuesta A, Silva CKRT, Silva SD (2021). Atenção ao parto por enfermeira obstétrica em maternidades
vinculadas à Rede Cegonha, Brasil - 2017. Ciênc Saúde Colet.

[13] Santos NCP, Vogt SE, Duarte ED, Pimenta AM, Madeira LM, Abreu MNS (2019). Fatores associados ao baixo Apgar em recém-nascidos em centro de
parto. Rev Bras Enferm.

[14] Medina ET, Mouta RJO, Carmo CN, Theme MM, Leal MC, Gama SGN (2023). Boas práticas, intervenções e resultados: um estudo comparativo
entre uma casa de parto e hospitais do Sistema Único de Saúde da Região
Sudeste, Brasil.. Cad Saúde Pública.

[15] Brocklehurst P, Hardy P, Hollowell J, Linsell L, Macfarlane A, Birthplace in England Collaborative Group (2011). Perinatal and maternal outcomes by planned place of birth for
healthy women with low risk pregnancies: the Birthplace in England national
prospective cohort study.. BMJ.

[16] Vogt AE, Diniz SG, Tavares CM, Santos NCP, Schneck CA, Zorzam B (2011). Características da assistência ao trabalho de parto e parto em
três modelos de atenção no SUS, no Município de Belo Horizonte, Minas
Gerais, Brasil. Cad Saúde Pública.

[17] Sandall J, Soltani H, Gates S, Shennan A, Devane D (2016). Midwife-led continuity models versus other models of care for
childbearing women. Cochrane Database Syst Rev.

[18] Barros GM, Dias MAB, Gomes SCS (2018). O uso das boas práticas de atenção ao recém-nascido na primeira
hora de vida nos diferentes modelos de atenção ao parto.. Rev Soc Bras Enferm Pediatras.

[19] Scarf VL, Rossiter C, Vedam S, Dahlen HG, Ellwood D, Foster D (2018). Maternal and perinatal outcomes by planned place of birth among
women with low-risk pregnancies in high-income countries a systematic review
and meta-analysis. Midwifery.

[20] Leal MC, Esteves-Pereira AP, Bittencourt SA, Domingues RMSM, Theme MM, Leite TH (2024). Protocolo do Nascer no Brasil II: Pesquisa Nacional sobre Aborto,
Parto e Nascimento.. Cad Saúde Pública.

[21] Ministério da Saúde Plataforma Integrada de Vigilância em Saúde. Painel de Monitoramento de
Nascidos Vivos; 2024..

[22] Leal MC, Pereira APE, Domingues RMSM, Theme MM, Dias MAB, Nakamura-Pereira M (2014). Intervenções obstétricas durante o trabalho de parto e parto em
mulheres brasileiras de risco habitual.. Cad Saúde Pública.

[23] Secretaria de Políticas de Saúde. Ministério da Saúde (2000). Programa de humanização do pré-natal e nascimento.

[24] Ministério da Saúde (2006). Pré-natal e puerpério: atenção qualificada e humanizada.

[25] World Health Organization (2018). WHO recommendations: intrapartum care for a positive childbirth
experience.

[26] Osório FL, Ayers S, Gonçalves F, Rocha JC (2021). City birth trauma scale-updates to the Portuguese
version. Arch Clin Psychiatr.

[27] Santos IS, Matijasevich A, Tavares BF, Barros AJD, Botelho IP, Lapolli C (2007). Validation of the Edinburgh Postnatal Depression Scale (EPDS) in
a sample of mothers from the 2004 Pelotas Birth Cohort Study. Cad Saúde Pública.

[28] Moreno AL, Sousa DA, Souza AMFLP, Manfro GG, Salum GA, Koller SH (2016). Factor structure, reliability, and item parameters of the
brazilian-portuguese version of the GAD-7 questionnaire. Temas Psicol.

[29] Baldisserotto ML, Theme MM, Griep RH, Oates J, Renó J, Cavalsan JP (2018). Transcultural adaptation to the Brazilian Portuguese of the
Postpartum Bonding Questionnaire for assessing the postpartum bond between
mother and baby.. Cad Saúde Pública.

[30] World Health Organization (2015). The prevention and elimination of disrespect and abuse during
facility-based childbirth.

[31] Bohren MA, Vogel JP, Hunter EC, Lutsiv O, Makh SK, Souza JP (2015). The mistreatment of women during childbirth in health facilities
globally a mixed-methods systematic review. PLoS Med.

[32] Ministério da Saúde; Agência Nacional de Vigilância
Sanitária (2008). Resolução RDC nº 36, de 3 de junho de 2008. Dispõe sobre
Regulamento Técnico para Funcionamento dos Serviços de Atenção Obstétrica e
Neonatal.. Diário Oficial da União.

[33] Aromataris E, Lockwood C, Porritt K, Pilla B, Jordan Z JBI manual for evidence synthesis..

[34] Comitê de Boas Práticas Científicas. Universidade de São
Paulo (2023). Guia de boas práticas científicas..

[35] National Academy of Sciences National Academy of Engineering
Institute of Medicine Committee on Science. Engineering. and Public
Policy (2009). On being a scientist: a guide to responsible conduct in
research..

[36] von Elm E, Altman DG, Egger M, Pocock SJ, Gøtzsche PC, Vandenbroucke JP (2008). The Strengthening the Reporting of Observational Studies in
Epidemiology (STROBE) statement guidelines for reporting observational
studies. J Clin Epidemiol.

[37] Ministério da Saúde (2022). Diretriz nacional de assistência ao parto normal: versão
preliminar.

